# The Butterfly Effect: Mild Soil Pollution with Heavy Metals Elicits Major Biological Consequences in Cobalt-Sensitized Broad Bean Model Plants

**DOI:** 10.3390/antiox11040793

**Published:** 2022-04-18

**Authors:** Raimondas Šiukšta, Vėjūnė Pukenytė, Violeta Kleizaitė, Skaistė Bondzinskaitė, Tatjana Čėsnienė

**Affiliations:** Institute of Biosciences, Life Sciences Centre, Vilnius University, Saulėtekio Ave. 7, LT-10322 Vilnius, Lithuania; vejune.pukenyte@gmc.vu.lt (V.P.); violeta.kleizaite@gf.vu.lt (V.K.); skaiste.bondzinskaite@gf.vu.lt (S.B.); egle.cesniene@gf.vu.lt (T.Č.)

**Keywords:** antioxidant response, CDDP polymorphism, Co pretreatment, soil pollution, transcript-based profiling, *Vicia faba*

## Abstract

Among the heavy metals (HMs), only cobalt induces a polymorphic response in *Vicia faba* plants, manifesting as chlorophyll morphoses and a ‘break-through’ effect resulting in the elevated accumulation of other HMs, which makes Co-pretreated broad bean plants an attractive model for investigating soil pollution by HMs. In this study, Co-sensitized *V. faba* plants were used to evaluate the long-term effect of residual industrial pollution by examining biochemical (H_2_O_2_, ascorbic acid, malondialdehyde, free proline, flavonoid, polyphenols, chlorophylls, carotenoids, superoxide dismutase) and molecular (conserved DNA-derived polymorphism and transcript-derived polymorphic fragments) markers after long-term exposure. HM-polluted soil induced a significantly higher frequency of chlorophyll morphoses and lower levels of nonenzymatic antioxidants in Co-pretreated *V. faba* plants. Both molecular markers effectively differentiated plants from polluted and control soils into distinct clusters, showing that HMs in mildly polluted soil are capable of inducing changes in DNA coding regions. These findings illustrate that strong background abiotic stressors (pretreatment with Co) can aid investigations of mild stressors (slight levels of soil pollution) by complementing each other in antioxidant content reduction and induction of DNA changes.

## 1. Introduction

Intensive urbanisation and industrialization processes together with transportation emissions are the main sources of environmental pollution, especially in populous areas [1,2,3]. Among pollutants, heavy metals (HMs) are especially important because of their long biological half-lives and high toxicity, they cause various physiological, biochemical, and molecular changes in plants, resulting in reduced growth, lower biomass production, ROS accumulation, and DNA damage [4,5,6]. HMs tend to accumulate in soil, in which the persistence of HMs is much longer than that in other components in the environment [7,8]. HMs can enter the food chain directly from the soil or leak into waters [9] and occur in the form of fine particles that can be inhaled and cause various human diseases [10,11,12]. Previous studies have shown the link between environmental genotoxicity assessed by plant tests and pollution-influenced human diseases [13]. The problem of heavy metal pollution is even more aggravated in former industrial territories which have been intensively transformed into living areas due to the rapid expansion of cities; in these areas, the contaminated soils are being cleaned or even replaced [14]. Nevertheless, continuous environmental monitoring data show that residual levels of HM pollution persist for decades [15,16]. However, the use of traditional soil chemical analysis has narrow applications in these environments, as it neither allows prediction of the biological effects of HMs nor indicates their bioavailability nor forecasts possible elemental interactions, which together make it necessary to employ biological tests in such cases [17,18].

Among tests in eukaryotes, various model plants, including *Allium cepa*, *Tradescantia*, *Crepis capillaris*, *Hordeum vulgare*, and *Vicia faba*, are considered more sensitive to pollution than model animals, making them particularly suitable for environmental biomonitoring studies [19,20,21]. Among these *A. cepa* and *V. faba* are approved and accepted as models by regulatory bodies for testing genotoxicity [22,23]. The *V. faba* root assay has been widely exploited to evaluate the genotoxicity of heavy metals [24,25,26], nanoparticles [27,28], and various organic chemicals [29,30]. Our previous research with *V. faba* revealed a unique excess Co-induced polymorphic response resulting in various degrees of chlorosis, the severity of which depends on the internal Co concentration [31,32]. Such a degree of variation in chlorophyll content was not observed after seed treatment with another sixteen heavy metals nor was the unique ‘break-through’ effect a simultaneous increase in the concentration of some heavy metals in leaves of chlorotic plants after seed pretreatment with Co salts [31]. This ‘break-through’ phenomenon was employed in the present study, which aimed to verify the suitability of Co-sensitized *V. faba* as a model for the detection of technogenic soil pollution with heavy metals. The experiments were conducted using a collection of soils containing various environmentally realistic heavy metal concentrations, which were collected from the territories of the closed factories ‘Grąžtai’ (1957–2007, which produced drills) and ‘Rimeda’ (1925–1992, formerly ‘Electrit’, which produced radio receivers and radio-measuring instruments), areas which are currently being used for housing estates and small enterprises. The complex soil pollution-induced effects in *V. faba* were investigated at the morphological, biochemical, and molecular levels by evaluating the frequency of chlorophyll morphoses, generation of reactive oxygen species (ROS), response of various antioxidant systems, and polymorphism spectra of transcripts and coding sequences of DNA. Additionally, the Co-sensitized *V. faba* model was used for the first time for the detection of soil pollution.

## 2. Materials and Methods

### 2.1. Soil Sample Characteristics

Soil samples were collected in Vilnius urban areas that were previously sites of the industrial factories ‘Grąžtai’ and ‘Electrit’. To cover as much of the former factory area as possible (approximately 4 ha), soil samples were collected from eight different points. Drill factory (DF) soil contamination and the multielemental geoindices Zs (soil total contamination index) and RI (potential ecological risk index) were determined as described previously [33].

### 2.2. Experimental Design

The experiments were conducted in 3 stages, beginning with the quantitative parameter analysis, for which only deionized water-imbibed *V. faba* beans were grown in eight different DF soils (DF1–DF8) with different pollution levels, and plant height, leaf number and dry weight were measured. At the second stage, cobalt (Co(NO_3_)_2_)-pretreated seeds were sown in the selected DF soils, contrasting in heavy metal content and composition, and germination and frequency of different groups of chlorophyll morphoses were counted. The third stage of the experiments included a combination of biochemical and molecular biomarkers. In this stage, only two types of soil were used: uncontaminated (control) and polluted DF soil, a mixture of newly collected soils from the DF territory. After four weeks of growth, the third leaves of Co-pretreated and untreated plants were collected for RNA and DNA extraction and biochemical analysis.

### 2.3. Induction of Chlorophyll Morphosis in Vicia faba Plants with Co(NO_3_)_2_

The (geno)toxic effect of DF soil was investigated using the *Vicia faba* cv. ‘Aušra’ chlorophyll morphosis model system under cobalt stress. *V. faba* cv. ‘Aušra’ was created from local varieties in the Institute of Agriculture, a department of the Lithuanian Research Centre for Agriculture and Forestry and is now preserved and propagated in the Botanical Garden of Vilnius University, from which this genotype was obtained for our study. To induce chlorophyll morphoses, broad bean seeds were soaked in 7.5 mM Co(NO_3_)_2_ solution for 12 h, control seeds were soaked in deionized water. Only fully swollen seeds were planted in uncontaminated (control) and polluted (DF) soil. After four weeks of growth, Co-treated plants were sorted according to their phenotypes into normal green (NG), light green (LG), and yellow (Y) groups, which were used for subsequent analyses.

### 2.4. Evaluation of Oxidative Stress in Plants Using Biochemical Markers

Colorimetric assays were used to evaluate a set of eight biochemical markers: hydrogen peroxide (H_2_O_2_), ascorbic acid (AscA), malondialdehyde (MDA), free proline, flavonoid, polyphenols and content of chlorophylls (Chl) a and b, and total carotenoids (Car). Biochemical analysis was performed in at least three independent replicates, with at least three biological replicates per each.

H_2_O_2_ content was determined using the Velikova and Loreto [34] protocol. An amount of 50 mg of leaf tissue was homogenized on ice in 0.5 mL of 0.1% trichloroacetic acid (TCA). The homogenate was centrifuged at 12,000 rpm for 15 min. Afterwards, 250 μL of supernatant was mixed with 250 μL of 10 mM potassium phosphate buffer (pH 7.0) and 500 μL of 1 M KI solution and absorbance was measured at 390 nm. The concentration of H_2_O_2_ was calculated as μM per g of fresh weight (μM/g FW) using a calibration curve.

The MDA level was determined according to Hodges et al. [35]. An amount of 100 mg of leaf tissue was homogenized in 2.5 mL of 80% ethanol and then centrifuged at 5000 rpm for 10 min. Then, 1 mL of the supernatant was added to one of two pre-prepared tubes: the first contained 1 mL of 20% TCA solution without thiobarbituric acid (TBA− solution), and the second—1 mL of 20% TCA and 0.65% TBA (TBA+ solution). Samples were then mixed and placed in a water thermostat for 25 min at + 95 °C. After that, the samples were suddenly cooled in an ice bath and centrifuged at 5000 rpm for 10 min. The optical absorbance of both solutions was then measured at 440 nm, 532 nm, and 600 nm. The concentration of MDA was calculated according to the formulas:

((Abs 532_TBA+_) − (Abs 600_TBA+_) − (Abs 532_TBA−_ − Abs 600_TBA−_)) = A (1)

((Abs 440_TBA+_ − Abs 600_TBA+_) × 0.0571) = B (2)

MDA (nmol/mL) = (A − B/157,000) × 10^6^ (3)

AscA content in plant leaves was determined according to Mitsui and Ohta [36]. An amount of 60 mg of leaf tissue was homogenized in 1 mL of ice-chilled 5.5% metaphosphoric acid and then centrifuged at 12,000 rpm, +4 °C for 10 min. Next, 250 μL of supernatant was mixed with 500 μL of 2% sodium molybdate, 500 μL of 0.15 N sulfuric acid, and 250 μL of 1.5 mM Na_2_HPO_4_ solution. In all steps, all the solutions were kept in an ice bath. Then the mixture was incubated at +60 °C for 40 min. Afterwards, the solution was rapidly cooled on ice, and optical absorbance was measured at 660 nm. The level of AscA (μM/g FW) was calculated using a calibration curve.

Free Pro content in plants was determined using the Ábrahám et al. [37] protocol. An amount of 100 mg of leaf tissue was homogenized in 500 μL 3% sulfosalicylic acid and then centrifuged at 13,500 rpm for 5 min. Then, 200 μL of supernatant was transferred into tubes containing 100 μL of sulfosalicylic acid, 200 μL of glacial acetic acid, and 200 μL of acidic ninhydrin (1.25 g ninhydrin dissolved in 30 mL glacial acetic acid and 20 mL of 6 M orthophosphoric acid). Afterwards, the reaction mixture was incubated at +96 °C for 60 min, and the reaction was terminated on ice. Next, 1 mL of toluene was added to the tubes, vortexed for at least 20 s, and optical absorbance was measured at 520 nm. Calculation of free Pro concentration (μg/g FW) was performed using a calibration curve.

To determine the content of polyphenols and flavonoids in the leaves, methanolic plant extracts were prepared according to Chew et al. [38]. An amount of 60 mg of fresh leaf tissue was homogenized in 3 mL 75% methanol and shaken at 200 rpm in the dark for 4 h. Afterwards, samples were centrifuged at 5000 rpm for 10 min and stored at –20 °C until further analysis.

For polyphenol content determination, 100 μL of methanol extract was mixed with 500 μL of 10% Folin–Ciocalteau phenolic reagent and 400 μL of 7.5% Na_2_CO_3_. Then mixtures were incubated in the dark for 30 min, and optical absorbance was measured at 765 nm. Total polyphenolic content (mg/g FW) in the samples was calculated with a calibration curve using gallic acid as a standard.

To determine total flavonoid content, 100 μL of methanol extract was mixed with 300 μL of 75% methanol, 20 μL of 10% AlCl_3_, 20 μL of 1 M sodium acetate, and 560 μL deionized H_2_O. The optical absorbance of the solution was then measured at 435 nm. Total flavonoid content (mg/g FW) in the samples was calculated with a calibration curve using quercetin as a standard.

Chl a, b and Car content in plants was measured according to Minocha et al. [39]. An amount of 30 mg of leaf tissue was placed in a tube containing 2 mL of N,N-dimethylformamide (DMF). The pigment extraction was performed in the dark at +4 °C for 72 h. Samples then were thoroughly mixed and centrifuged for 5 min 13,500 rpm, and optical absorption was measured at 480 nm, 647 nm, and 664 nm. The content of Chl a, b and Car was calculated according to the formulas and converted to mg/g FW:

Chl a = (12 × A664) − (3.11 × A647)(4)

Chl b = (20.78 × A647) − (4.88 × A664)(5)

Car = (1000 × A480 − 1.12Chl a − 34.07Chl b)/245(6)

Superoxide dismutase (SOD, EC 1.15.1.1) polymorphism was analysed from the second or third upper leaf of individual plants. An amount of 1 g of leaf tissue was homogenized in 2 mL of chilled 50 mM potassium phosphate buffer (pH 7.8). The homogenate was centrifuged at +4 °C, 12,000 rpm for 15 min. Native polyacrylamide gel electrophoresis (PAGE) and enzyme activity staining were performed according to Kleizaite et al. [40].

### 2.5. DNA Extraction and CDDP Analysis

Genomic DNA was extracted following a modified procedure described by Doyle and Doyle [41] from plants after four weeks of growth in control soil and polluted DF soil mix. PCR was performed as described by Collard and Mackill [42]. For the screening for DNA polymorphisms, five previously selected primers (WRKY-R1, Myb2, ERF1, KNOX3, ABP 1-1) were included in the analysis.

### 2.6. Transcript Profiling Using Differential Display Technique

Transcript profiling by the differential display method was performed according to Šiukšta et al. [43]. Briefly, total RNA was extracted from the third leaf of *V. faba* plants using the innuPREP Plant RNA Kit and innuPREP DNase I Digest Kit (Analytik Jena, Überlingen, Germany) following the manufacturer’s recommendations. The reverse transcription and differential display PCR with 24 primer combinations, resulting in transcript-derived fragments (TDFs), were performed using RNAimage Kit 6 (GenHunter, Nashville, TN, USA) according to the manufacturer’s protocol. Amplification products were separated by electrophoresis on 6% denaturing polyacrylamide gels in 1× TBE buffer and stained with silver according to Benbouza et al. [44]. Polymorphic TDF bands were cut out and extracted from the gel by boiling method, reamplified in two rounds of PCR, purified using Zymoclean Gel DNA Recovery Kit (Zymo Research, Irvine, CA, USA), and sequenced by BaseClear (Leiden, The Netherlands). 

### 2.7. CDDP- and TDF-Based Dendrogram Construction

CDDP fingerprinting and transcript profiling procedures using the differential display technique were performed in at least two independent replications, and only reproducible CDDP or TDF bands were used to make a binary matrix for further analysis. Nei and Li [45] genetic distance-based UPGMA dendrograms were constructed using TREECON v. 1.3b software [46]. Homology searches for the sequenced polymorphic TDFs were performed using the BLAST algorithm [47] in the NCBI database.

### 2.8. Statistical Analysis

All the results are representative of three independent experiments performed at least in triplicate, and in the tables and figures, results are expressed as mean or percentage (%) ± standard error (SE) of the mean. The data normality was tested using the Shapiro–Wilk test using Past (version 3.26) software. As data did not follow a normal distribution, a nonparametric Kruskal–Wallis rank-sum (omnibus) test was performed using Statistica 64 (version 12.0) software, followed by the Dunn’s test with Benjamini–Hochberg (FDR) adjustment, used as a post hoc test. To compare the percentages, the two proportion Z-test analysis was conducted using Microsoft Excel (Version 2202 Build 16.0.14931.20128).

## 3. Results

### 3.1. Chemical Characteristics of the Polluted Soil Samples

The territory of the former drill factory (DF), in which soil sampling was performed, is heavily contaminated by a range of potentially harmful elements, such as Cd, Cr, Hg, Mo, Pb, and Zn (Table 1), that exceed the control and Lithuanian Hygienic Norm HN 60:2015 values to various extents [33]. 

In each of the investigated soil samples (DF1–DF8 and DF soil mix), a different set of potentially harmful elements was identified. Only two samples (DF3 and DF7) exhibited excessive amounts of Cd, three samples (DF1, 6, and 7) exhibited excessive amounts of Pb, three samples (DF2, 6, 7) exhibited excessive amounts of Zn, and only DF6 had an excess of Hg. Based on the Zs (soil total contamination index) value, all soil samples could be differentiated into the average hazard or hazard categories, but the RI (potential ecological risk index) value marked all samples as low risk. The highest Zs values were determined in DF6 (Zs = 104) and DF7 (Zs = 83), which were the richest samples with respect to heavy metal composition, and the lowest Zs values were determined in DF4 (Zs = 17), in which none of the elements exceeded the limits according to Lithuanian Hygienic Norm HN 60:2015. DF soil mix, which was newly collected from more sites of the DF territory and used in the third stage of the experiments, could be considered hazardous according to the Zs values since Cr and Zn concentrations in the DF soil mix exceeded the norm. Co concentration in all the tested soil samples did not exceed the norm and varied in the range of 3–17 ppm in DF soil samples and was 7 ppm in control soil. Full chemical analysis of the soil samples DF1–8 was published previously by Čėsnienė et al. [33].

### 3.2. The Effect of Polluted Soils on Quantitative Parameters of V. faba Plants

The first stage of the experiment aimed to roughly evaluate whether (and how) some easily measured quantitative parameters (plant height, leaf number per plant, and dry weight) of the *V. faba* model system are responsive to the soil pollution level, as evaluated through the multielemental geoindices Zs and RI. The abovementioned parameters of plants subjected to polluted soils for one month are presented in Table S1A.

As expected, the highest values of all tested quantitative features were determined in plants grown in control soil (Table S1). Quite a paradoxical situation was observed after a comparison of plants grown in the most and least polluted DF soils (DF6 and DF4, respectively) when all tested parameters exceeded the average values of DFs but only insignificantly differed from each other. To test the possible relationships between the quantitative parameters and soil geochemical indices, Pearson correlation analysis and principal component analysis (PCA) were performed (Table S1B and Table S1C, respectively). Naturally, a strong positive correlation was observed between all three quantitative plant characteristics, but only a weak or no correlation was determined between quantitative plant parameters and Zs or RI. However, Zs and RI were strongly positively correlated with each other (Table S1B).

**Figure 1:**
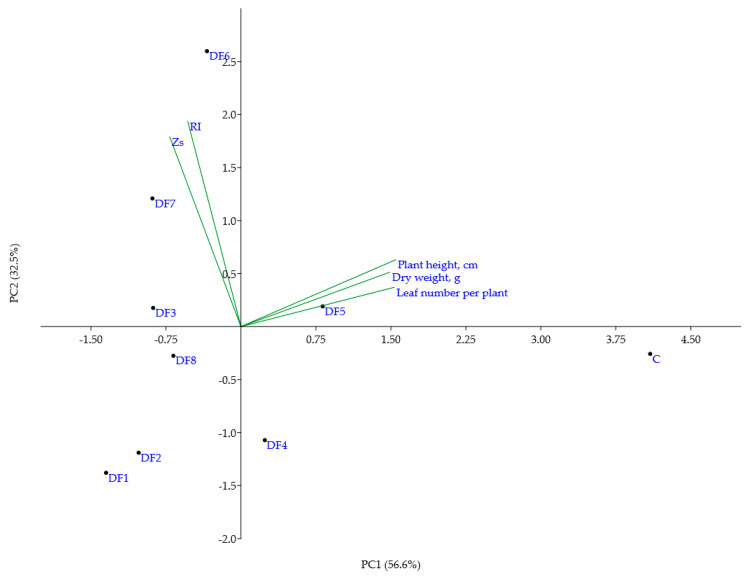
Results of PCA using quantitative characteristics of broad bean plants grown in polluted soils with different extents of heavy metal pollution. DF1–DF8—polluted soil from different sites of the drill factory.

The PCA biplot (Figure 1), generated using the first two principal components, both explaining 89.1% of the total variance (Table S1C), showed that even plants grown in DF soils, differing in Zs values almost two-fold and in RI values almost six-fold (e.g., DF3 and DF8), grouped closely as well as plants from DF soils possessing almost the same Zs and RI values (e.g., DF1 and DF2). PC1 was moderately correlated with all quantitative parameters of plants, while PC2 showed a moderate correlation with soil geoindices (Table S1C). In general, the abovementioned data analysis showed that all soil samples from the polluted DF area had a strong suppressive effect on the tested quantitative parameters of *V. faba* plants, but such an effect only scarcely depended on the intensity of soil pollution, leading to the conclusion that the selected characteristics of *V. faba* plants are not adequate indicators for the simple evaluation of soil pollution status.

### 3.3. The Use of Co-Induced Chlorophyll Morphoses for the Evaluation of Soil Pollution

**Figure 2:**
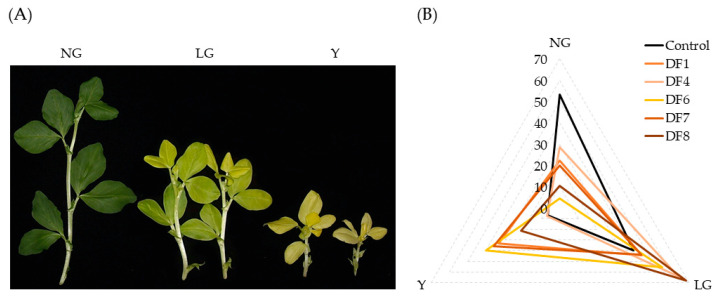
Phenotypic groups (**A**) and frequencies (**B**) of Co-induced chlorophyll morphoses (NG—normal green, LG—light green, Y—yellow) observed in *V. faba* plants grown in soil possessing different pollution levels. DF1,4,6,7,8—polluted soil from different sites of the drill factory.

Since the first stage of the experiments showed a poor relationship between the soil pollution level and the response of *V. faba* quantitative parameters, in the second stage of the experiments, we included seed priming with 7.5 mM Co(NO_3_)_2_ solution, which is known to induce chlorophyll morphoses in *V. faba* plants. The spectrum of Co-induced chlorophyll morphoses covered plants with unchanged (normally green, NG), partially changed (light green, LG), and fully changed (yellow, Y) phenotypes (Figure 2A). After one month of growth in different DF soil samples, the germination rate and frequency of each phenotypic group were scored (Figure 2B and Table S2A). The germination rate among the DFs ranged from 83% to 95%, with the lowest value observed in the DF7 soil (Table S2A). Among others, the rarest group of Co-treated plants was the most severe phenotype (Y), ranging from 7% in control soil to 40% in DF6, and a significant increase in the frequency of the Y group was observed in almost all tested soils (Table S2A). However, the increase in the percentage of the Y and LG groups occurred mainly with the decrease in the frequency of NG plants (Figure 2B).

Correlation analysis of data from the second stage of the experiment revealed moderate negative correlations between the soil geoindices and germination rate and moderate or strong negative correlations between the RI or Zs and the frequency of NG plants, respectively (Table S2B). In contrast, RI or Zs showed a strong positive correlation with each other and with the frequency of the most severe phenotype of Co-induced morphoses (Y group).

**Figure 3:**
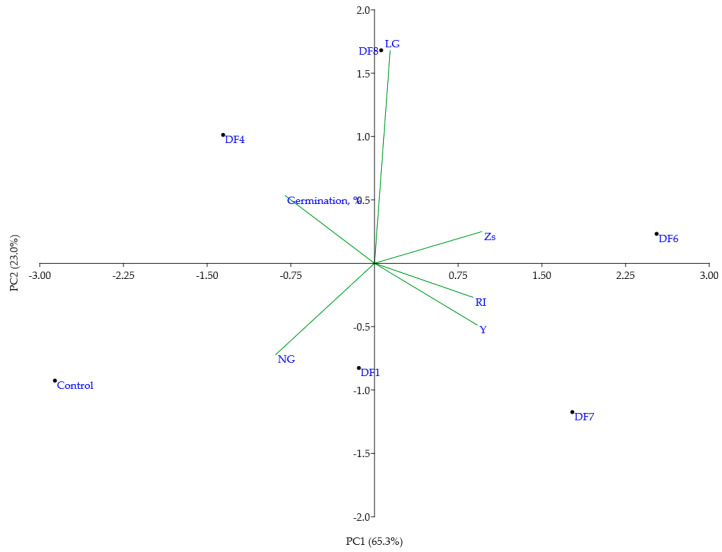
Biplot of the first two principal components (PCs) (accounting for 88.3% of the variance) obtained by including two chemical analysis-based soil indices (Zs and RI), germination rate, and frequencies of Co-induced morphoses in *V. faba* plants grown in soils with different industrial pollution levels. Phenotypes of morphoses: NG—normal green, LG—light green, Y—yellow; C—plants grown in control soil; DF1,4,6,7,8—plants grown in soil collected from different sites of the drill factory.

To summarize the complete data from the second stage of the experiment, a multivariate technique (PCA) was used (Figure 3). Together, PC1 and PC2 explained 88.3% of the total variance (Table S2C). PC1 was moderately positively correlated with the soil geochemical indices and percentage of Y plants and negatively correlated with the germination rate and frequency of Co-treated NG plants, while PC1 was strongly positively correlated with Co-treated light green (LG) plants. In the PCA biplot, the most distant from the control soil were DF6 and DF7, which possessed the highest geoindices (Zs = 104 and 83, RI = 128.74 and 83.16, respectively), while soil samples with intermediate RI and Zs values (DFs 1, 4, and 8) were positioned between the most polluted and control soils (Figure 3). Altogether, the data from the second stage of the experiment showed that the Co-sensitized *V. faba* model system reacted to soil contamination more consistently than untreated plants and therefore was suitable for the analysis of soil contamination-induced effects.

### 3.4. The Biochemical Response of Co-Treated V. faba Plants to Mild Heavy Metal-Induced Stress

**Figure 4:**
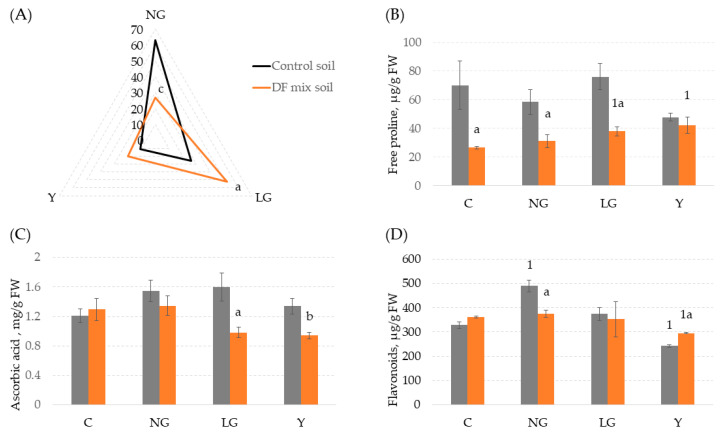
Distribution of phenotypic groups (**A**) and content of free proline (**B**), ascorbic acid (**C**), and flavonoids (**D**) in Co-pretreated *V. faba* plants after one month of exposure to the mildly polluted soil mix. Phenotypes of morphoses: NG—normal green, LG—light green, Y—yellow; C—plants grown in control soil; DF—plants from soil collected from different sites of the drill factory. Significance level: ^a^ *p* < 0.05, ^b^ *p* < 0.01, ^c^ *p* < 0.001 compared with the respective phenotypic group from control soil; ^1^ *p* < 0.05, compared with Co-untreated plants grown in the same soil variant.

During the third stage of the experiment, more numerous populations of Co-treated *V. faba* plants were grown in control soil and DF soil mix (Figure S1), collected from more sites of the drill factory territory to include a panel of biochemical stress markers in the analysis. After 1 month of growth, a significant increase (*p* < 0.05) in the frequency of Co-treated plants with the LG phenotype in the DF soil mix was observed at the expense of a significant decrease (*p* < 0.001) in the percentage of plants with an unchanged phenotype (NG) (Figure 4A).

To obtain a comprehensive view of polluted soil-induced changes in *V. faba* plants at a biochemical level, three groups of biomarkers were included in the analysis: (I) concentrations of MDA and H_2_O_2_, which were expected to reflect the extent of oxidative stress-induced damage; (II) enzymatic (SOD) and nonenzymatic (free Pro, AscA, polyphenols, and flavonoids) antioxidant systems, used as markers of the stress response; and (III) the content of the main photosynthetic pigments (Chl a, Chl b, Car), which determines the general photosynthetic capacity of plants. A complete set of results of all tested biochemical markers is presented in Tables S3A–C.

Considering the biomarkers from Group I, the concentration of H_2_O_2_ was found to be approximately the same in all plant groups grown in both control and DF mix soils, while the MDA content significantly decreased in some groups of Co-treated plants (NG plants from control soil and all Co-treated plants from DF soil mix independent of the plant phenotype) but in an irregular manner (Table S3A, Group I). The concentration of polyphenols was significantly higher in all phenotypic groups of Co-treated plants grown in control soil, but in all Co-treated plants from the polluted DF soil mix, such a response was absent (Table S3A, group II).

Only some of the nonenzymatic antioxidants from group II (concentrations of free Pro, AscA, and flavonoids) could be considered soil pollution-influenced biomarkers (Figure 4 and Table S3A). The variation in the content of free Pro among the Co-treated and untreated plants grown in the control soil was insignificant, but in the Co-treated plants grown in the contaminated DF soil mix, the free Pro concentration increased in a phenotype severity-dependent manner and was significantly higher (*p* < 0.05) in the LG and Y plant groups than in the Co-untreated (control) plants (Figure 4B). Interestingly, the level of free Pro was lower (*p* < 0.05) in plants grown in the polluted DF soil mix than in plants from the control soil, with the exception of Y plants, in which the free Pro concentration did not depend on the soil pollution status.

Comparable levels of AscA were determined in *V. faba* plants of unchanged phenotype (C and NG groups) independent of soil type, while in DF soil mix-grown plants possessing chlorophyll morphoses (LG and Y groups), the concentration of AscA was significantly reduced compared with that of the corresponding plant groups from the control soil (Figure 4C). Notably, the concentrations of AscA remained unchanged in all groups of Co-treated plants grown in control soil, independent of the phenotypic severity of the plants.

The concentrations of flavonoids in Co-treated plants were either phenotype- or soil pollution status-dependent (Figure 4D): an increased level of flavonoids was observed only in NG plants from control soil, while significantly decreased flavonoid concentrations were observed in plants with the most pronounced chlorophyll morphoses (Y group) grown in both control and DF mix soils. Other tested nonenzymatic biochemical markers showed irregular patterns of variation between Co-treated plant phenotypic groups and/or soil pollution status (Table S3A–C). In addition, significant positive correlations were found between the content of most of the tested nonenzymatic antioxidants (AscA and polyphenols/free Pro, Car, and flavonoids) (Table S3D).

Our study revealed the presence of the three types of SOD isozyme profiles in *V. faba* plants, with the main profile having five SOD electrophoretic bands (Table S3B). The reaction with KCN showed two SOD bands (Mn-SOD and Fe-SOD) in all tested samples. Among Cu/Zn-SOD, three SOD isoforms (a, b, and c) were detected. The ratio of SOD isoenzyme profiles in *V. faba* plants was found to be comparable in Co-untreated (control) and Co-treated NG plants, in which the least common was SOD profile type II, regardless of soil type. Interestingly, in LG plants grown in the DF soil mix, the frequency of the SOD type II profile was increased, while in LG plants from control soil, not only the frequency of type II but also the frequency of type III decreased. The most similar ratio of SOD profiles was observed in Co-treated Y plants grown in both soil types.

The decrease in the concentrations of the photosynthetic pigments (chlorophylls a, b, and carotenoids) in the Co-treated plants was phenotype severity dependent, but no significant differences were observed between the respective plant groups grown in the control and DF soil mix (Table S3C). Not surprisingly, strong significant correlations were detected not only between the concentrations of Chl a, Chl b, and Car but also between the ratios of Chl a/b and Chl/Car (Table S3D).

**Figure 5:**
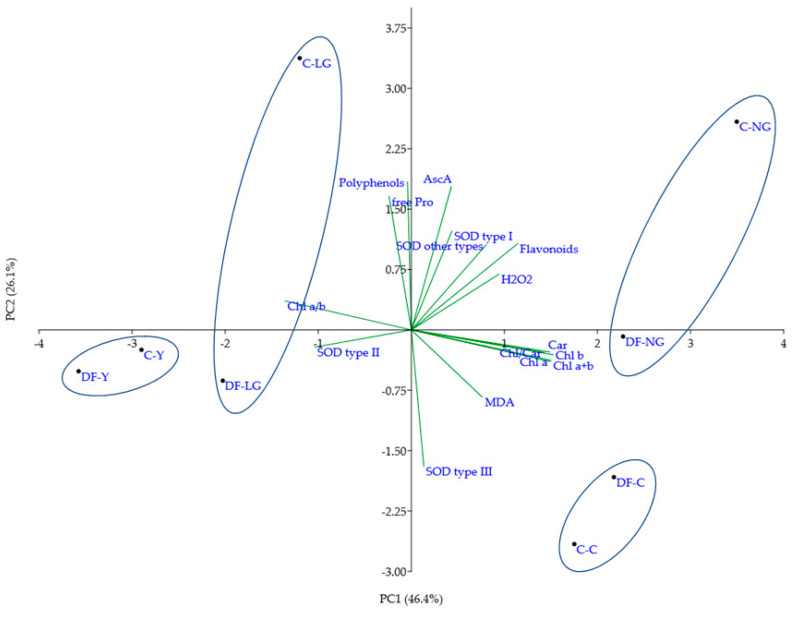
Biplot representing the first two principal components (PCs) (accumulating 72.5% of variance) generated using all tested biochemical parameters (ROS generation, antioxidants, and photosynthetic pigments). Ellipses enclose the same treatment and phenotypic groups of *V. faba* plants grown in control and polluted soils. Phenotypes of morphoses: NG—normal green, LG—light green, Y—yellow; C—plants grown in control soil; DF—plants from soil collected from different sites of the drill factory.

To generalize the overall response of the tested biochemical markers to heavy metal stress, principal component analysis (PCA) was performed (Figure 5). The first two principal components (PC1 and PC2) together explained the majority (72.5%) of the total variance, so they were included in the subsequent analysis (Table S3E). PC1, which explained the majority of the total variance (46.4%), was correlated mainly with the content of photosynthetic pigments (Chl a, b, and carotenoids) and some biochemical markers (H_2_O_2_, flavonoids, and SOD profile type II), which differentiated plants according to the phenotypes of morphoses independent of the soil pollution status, grouping individuals with normal (control and Co-treated NG) and lower (LG and Y groups) pigment content separately (Figure 5). PC2 was mostly correlated with the content of most tested antioxidants (free Pro, AscA, polyphenols, flavonoids, and SOD profile types I and III) and divided the plant phenotypic groups according to the soil type in which they were grown (Table S3E). Based on the whole set of biochemical data, Co-untreated (control) plants from control and polluted DF soil (C-C and DF-C, respectively) grouped as well as Co-treated plants of the most severe phenotypes (C-Y and DF-Y), showing the effect of soil pollution status to have a relatively mild effect on the biochemical profiles of these phenotypical groups (Figure 5). Quite an opposite situation was observed in the case of Co-treated plants of LG and even NG (unchanged) phenotypes, two major groups of Co-induced morphoses, which markedly differed in their biochemical spectra depending on soil pollution status (Figure 5): NG plants from control and DF soils were located distantly on different sides of the PC2 axis (quadrants I and IV, respectively); a comparably long distance also separated LG plants grown in both soil types (quadrants II and III, respectively). In general, NG and LG plants grown in control soil possessed higher content of the tested antioxidants in comparison with the respective groups of plants grown in the DF soil mix.

### 3.5. Evaluation of Heavy Metal-Induced Changes in V. faba Plants Using Molecular Markers

To evaluate the effects of polluted soil in *V. faba* plants at the RNA level and to reveal the nature of Co-induced morphoses, small-scale RNA fingerprinting using a differential display technique was performed. In total, 56 polymorphic transcript-derived fragments (TDFs) were excised from PAA gel, 31 of which were successfully reamplified by two-step PCR sequenced and used for sequence homology searches and TDF-based UPGMA clustering. The length of TDFs used in the following analysis varied in the range of 150–540 nt. A full list of the homology search results of all sequenced polymorphic TDFs is shown in Table S2. The results of the homology search of selected fourteen polymorphic TDFs are presented in Table 2 since they were shown to be soil- or plant phenotypic group-specific (Groups I and II, respectively).

Ten polymorphic TDFs were specifically found in plants grown in control soil (Group I; TDFs N4, 13, 18, 29, 33, 37/41, and 49) or polluted DF soil mix (group II; TDFs N17, 47, and 54) independent of Co treatment status and, consequently, the phenotypes of morphoses (Table 2). Interestingly, TDF N33 was detected in the control and Co-treated plants of unchanged phenotype (NG) grown only in unpolluted (control) soil. Polymorphic TDFs of group III were observed in plants grown in both control and DF soils, but their bands differed in the intensity depending on soil contamination level: TDF N35 was more pronounced in plants from control soil, while the intensity of TDF N36 was higher in plants grown in DF soil mix.

TDFs of group IV were found to be plant treatment- and phenotypic group-specific: TDF N32 was only found in TDF-derived profiles of Co-untreated plants, whereas N40 was characteristic of Co-treated plants with the highest suppression of chlorophyll biogenesis (Y phenotype) (Table 2).

**Figure 6:**
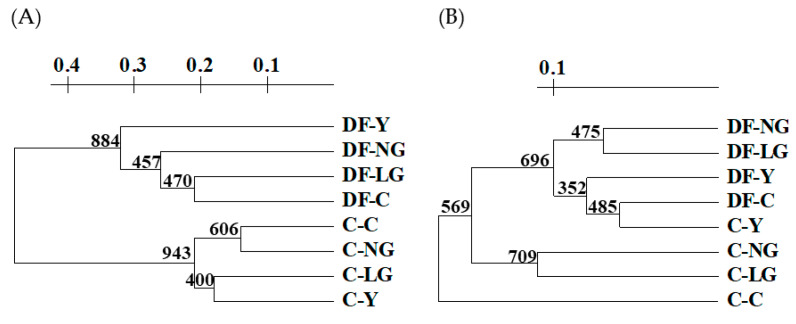
UPGMA dendrograms were generated using the conserved DNA-derived polymorphism (CDDP) (**A**) and variation in the profiles of transcript-derived fragments (TDFs) (**B**). The Nei and Li genetic distances are presented on the axis, and bootstrap values from 1000 iterations are shown at the branch nodes.

To reveal the relationship among the various phenotypic groups of Co-treated plants grown in clean and polluted soils, the UPGMA dendrogram using Nei and Li (1979) distances was constructed according to the binary pattern of sequenced polymorphic TDF-based fingerprints (Figure 6A). Two major clusters consisting of plants grown in (I) DF soil mix and (II) control soil can be distinguished: in the DF cluster, Co-treated Y plants formed the most distinct group from the remaining phenotypic groups of chlorophyll morphoses observed in DF soil, while in the cluster of control soil, plants with an unchanged chlorophyll content (Co-untreated (C) and Co-treated NG plants) grouped together as well as plants with various degrees of disturbed synthesis of photosynthetic pigments (LG and Y phenotypes) (Figure 6A).

In parallel to TDF-based fingerprinting, the evaluation of the Co- and polluted soil-induced damage was performed using functional CDDP markers directed towards the conserved DNA regions of the main families of transcription factors. In total, 78 loci were obtained with five CDDP primers, 36 of which (46.2%) were polymorphic (Table S5). The majority of polymorphic loci were generated with the ABP1-1 primer, which is complementary to the conserved region of plant-specific auxin binding proteins, whereas the least number of polymorphic CDDP loci was determined with the primer Myb2. The CDDP polymorphism-based UPGMA dendrogram shows that the genetic distances estimated with CDDP markers were very low. The most distinct clade was observed for Co-untreated plants grown in clean soil (Figure 6B). Two phenotypic groups of Co-treated plants (NG and LG) from the same (unpolluted) soil also branched off from the DF cluster as a distinct clade, indicating that the CDDP profiles of plants from unpolluted soil were more similar to each other than those of plants grown in the polluted DF soil mix. Interestingly, plants grown in the polluted DF soil mix fell into one cluster with the most severe phenotype of Co-treated plants (Y) from the control soil. Moreover, the latter was grouped with DF control (Co-untreated) plants, indicating DF soil to be sufficiently toxic to induce DNA variation, similar to that observed in Co-treated plants. In general, the topologies of the UPGMA dendrograms generated using TDF- and CDDP-based polymorphisms were the same: both methods were able to discriminate between plants grown in soils with different pollution modes (Figure 6A,B).

## 4. Discussion

This study aimed to evaluate the long-term effect of residual industrial pollution of soil using *Vicia faba* as a model system. The soils, varying in degrees of contamination, for the study were collected from multiple sites of the former drill factory, a large territory that was converted into a residential area in the 1970s. Even more than half a century after recultivation, the periodic monitoring of soil pollution in this area shows elevated concentrations of various heavy metals [33].

The present study consisted of three successive stages. The first phase of the study searched for possible correlations between soil pollution levels and *V. faba* quantitative parameters (plant height, leaf number and dry weight), which were selected for their well-known sensitivity to heavy metals [48,49,50,51,52]. At this stage of the study, all the contaminated soil samples were found to significantly inhibit the abovementioned plant parameters (Table S1), but the decrease in such characteristics was only weakly correlated with the soil pollution level. A lack of a direct relationship between the degree of environmental contamination and the biological response has also been demonstrated in some studies assessing environmental genotoxicity [53,54,55]. The reason leading to such an incongruence may lie in the especially complex chemical nature of such environmental samples, such as soil, water, and air containing unique ratios of individual elements, which makes the prediction of their biological effects fairly complicated. Moreover, many studies of the effects of metals on living systems are performed using artificial monometallic contamination, while the cumulative effects of two or more metals in planta are much less investigated [56,57,58,59]. On the other hand, the interpretation of the response to heavy metal cocktails is further complicated not only by the synergistic, antagonistic, and additive interactions of metal pairs [60] but also by the dependence of such interactions on the species [61] and even on the organ of the plant, when the interactions of the same bimetallic mixture in the above-ground and underground parts of the plant are different [56,59].

To make the *V. faba* model system more sensitive to soil pollution, a phenomenon of Co-induced chlorophyll morphoses, the unique and easily evaluated feature of *V. faba* plants, was utilized during the second stage of the experiments (Figure 2 and Figure 3, Table S2). Even though various heavy metals can lead to chlorosis in a variety of plant species [62,63], only seed treatment with relatively high doses of Co^2+^ out of 16 tested heavy metals in our previous study was shown to induce a polymorphic response concerning the chlorophyll content in *V. faba* [31]. In contrast, low doses of Co are essential to plants, especially to leguminous species, including *V. faba*, in which Co takes part in nodulation and nitrogen fixation [50,64,65,66]. After seed treatment with 7.5 mM Co(NO_3_)_2_, the resulting *V. faba* populations grown in all polluted soil samples consisted mainly of plants with changed chlorophyll content (light green and yellow groups), which was significantly positively correlated with both soil geochemical indices Zs and RI (Figure 2B and Table S2). Moreover, after pretreatment of *V. faba* seeds with Co, even soil samples with the lowest risk and hazard levels (e.g., DF1 and DF4; Tables S1A and S2A) were capable of inducing a statistically significant shift in the spectra of Co-induced morphoses, suggesting that the *V. faba* model system can serve as a sensitive bioindicator for relatively low levels of soil pollution.

To verify the proposed suitability of the Co-sensitized *V. faba* for the indication of low levels of metal contamination, the third series of experiments was conducted to determine the impact of DF soil mix with low potential ecotoxicological risk on the oxidative stress-reflecting biochemical and molecular markers (Table S3). Biochemical analysis of plants involved the evaluation of reactive oxygen species (ROS) generation, plant antioxidant response, and photosynthetic pigment evaluation. Contrary to expectations, the level of H_2_O_2_ remained unchanged in all phenotypic groups of Co-treated and even untreated (control) plants, whereas the MDA content decreased in all the phenotypic groups of Co-treated plants independent of the soil pollution status (Table S3). Moreover, the levels of all tested nonenzymatic antioxidants were significantly lower only in Co-treated plants grown in the polluted DF soil mix than in the respective phenotypes from the control soil, although the dynamics of individual antioxidant responses were phenotype dependent (Figure 4B–D and Table S3). Such a suppressive effect of DF soil mix on levels of ROS and antioxidants could be influenced by at least a few factors. The chemical analysis of the DF soil mix revealed elevated levels of Cr and Zn (Table 1), the latter of which is known as an essential micronutrient of plants [50,60]. Since the application of Zn was shown to effectively mitigate heavy metal stress in various species of plants by reduction in MDA and ROS content [67,68], the presence of a slightly elevated Zn amount in DF soil mix could have a beneficial effect, alleviating the toxic effect of other heavy metals found in soil, such as Cr. However, in parallel to soil-borne Zn and Cr, *V. faba* plants were subjected to the third heavy metal, Co, at the early stage of development; thus, the general response to at least three heavy metals in planta could be considered. Although the diversity of heavy metal interactions in binary mixtures exists, only additive or antagonistic effects seem to prevail, especially when heavy metals exceed the optimal concentrations [69]. However, metal interactions in tertiary and quaternary mixtures are mainly antagonistic [56,70], indicating that weak or even unpredictable common effects of all three heavy metals (Zn, Cr, and Co) entering the *V. faba* plants from different sources at different developmental stages (Co-treatment of seeds, chronic exposure to Zn and Cr throughout the growth period) should be taken into account.

On the other hand, the nature of the Co-sensitized *V. faba* model system can also significantly contribute to the reduced antioxidant levels observed in Co-treated plants grown in the polluted soil, as it directly dictated the experimental design. First, biochemical and molecular parameters in broad bean plants could have been assessed only after long-term exposure to heavy metal stress because one month of growth is needed for Co-treated plants to reach sufficient size for appropriate differentiation into distinct phenotypic groups (NG, LG, and Y) according to the degree of damage. Although the response and dynamics of individual antioxidants in the presence of various abiotic stimuli are highly stressor-, exposure time-, organ-, age-, and plant species-dependent [59,71,72,73], the common response tends to peak in the first few days or weeks of the exposure and then decrease [72,74,75,76], suggesting that the exposure period of one month, which was inevitable in the present study, could have been too long to reflect the antioxidant peak. Second, in addition to its role in the induction of chlorophyll morphoses, Co is also known as an inhibitor of ethylene biosynthesis [77], which together with other phytohormones plays a pivotal role in the plant response to heavy metal stress [78,79,80]. It was shown that the excessive treatment of heavy metal-stressed plants with Co does not lead to enhanced ethylene production [81,82], an essential factor for the induction of ROS generation and antioxidant response [79]; thus, *V. faba* seed pretreatment with Co in our study can at least partially explain the unexpectedly low levels of H_2_O_2_ and MDA as well as nonenzymatic antioxidants observed in Co-treated plants. It is noteworthy that the level of antioxidants in Co-treated plants in the control soil remained mostly at the same level as in Co-untreated (control) plants, indicating that seed pretreatment with Co did not induce a substantial effect on antioxidant balance, even in plants with the most severe yellow phenotype.

During the third stage of the experiments, the effect of Co-treatment and mild soil pollution in *V. faba* was also evaluated at the molecular level using transcript- and CDDP-based fingerprints. Surprisingly, both dendrograms showed quite a similar topology (Figure 6), allowing the differentiation of plant groups not only according to the phenotype severity of Co-treated plants but also by exposure to heavy metal pollution of soil, as two major clusters consisting of control- and polluted soil-grown plants could be distinguished. The soil- or plant phenotypic group-specific polymorphic TDFs (Table 2) were then analysed for their possible importance in the heavy metal stress response, but little was revealed in the literature about their relation to heavy metal-induced effects in plants. Nevertheless, it is well established that responses to metal stress are generally drought responses since exposure to metals as primary stress triggers drought as secondary stress [83,84,85]. In this regard, all the abovementioned polymorphic TDFs were found to participate in drought stress [86,87,88,89,90,91,92,93,94,95,96,97] (the order corresponds to Table 2), suggesting their role in the heavy metal stress response in plants as well.

Heavy metal-induced genotoxic effects can be detected by employing various strategies, including DNA fingerprinting techniques, among which ISSR and RAPD are predominant [54,55,98,99,100,101,102,103]. Both are mostly directed to noncoding DNA sequences that can be highly polymorphic but usually make little or no contribution to phenotype [104,105]. In contrast, the conserved DNA-derived polymorphism (CDDP), which was used in the present study for potential heavy metal toxicity evaluation, is a gene-targeted marker system that can easily generate functional markers related to a particular plant phenotype [42]. HM-induced DNA polymorphisms can be naturally expected in Co-treated *V. faba* plants, as high doses of Co are mutagenic, especially in plants [106]. Surprisingly, the CDDP technique also led us to discriminate even between Co-untreated (control) plants grown in clean and mildly polluted DF soils, showing that even relatively low concentrations of environmental metal toxicants are capable of inducing changes even in coding regions of DNA. To our knowledge, this study marks the first time that the gene-targeted CDDP marker system has been used for genotoxicity assessment.

## 5. Conclusions

Our study indicates that the model system of Co-induced chlorophyll morphoses in *Vicia faba* is highly sensitive to various degrees of heavy metal pollution at the biochemical, molecular, and morphological levels. Even a slight increase in heavy metal concentrations in the soil had a striking suppressive effect on antioxidant content, and under a reduced antioxidant background, it was sufficient to induce changes in coding regions of DNA. Taken together, the data of the present study suggest that in the presence of strong abiotic stressors (pretreatment with Co), small stressors (slight levels of soil pollution) can play a crucial role in plant ROS homeostasis, highlighting the importance of context when evaluating particular stress responses.

## Figures and Tables

**Table 1:** Heavy metal content and hazard/risk of soil samples collected from the recultivated territory of a former drill factory.

Soil Sample	Zs ^a^		RI ^b^		Elements Which Exceed Limits According to Lithuanian Hygienic Norm HN 60:2015 ^c^					
	Value	Hazard Level	Value	Risk						
C	-	-	2	low						
DF1	26	average hazard	12	low				Mo^1.1^	Pb^1.2^	
DF2	30	average hazard	11	low				Mo^1.5^		Zn^1.1^
DF3	37	hazard	80	low	Cd^1.9^					
DF4	17	average hazard	9	low						
DF5	65	hazard	14	low		Cr^1.1^		Mo^6.1^		
DF6	104	hazard	129	low		Cr^1.2^	Hg^2.8^	Mo^7.2^	Pb^1.3^	Zn^2.0^
DF7	83	hazard	83	low	Cd^1.6^	Cr^1.1^		Mo^4.6^	Pb^1.2^	Zn^1.7^
DF8	67	hazard	14	low		Cr^1.0^		Mo^6.6^		
DF soil mix	67	hazard	14	low		Cr^1.2^				Zn^1.1^

^a^ Zs—topsoil total contamination index; Z < 16—permissible, 16 < Z < 32—average hazard, 32 < Z < 128—hazard, Z > 128—extreme hazard. ^b^ RI—potential ecological risk index; RI < 150—low risk, 150 < RI < 300—moderate risk, 300 < RI < 600—high risk, RI ≥ 600—very high risk. ^c^ Maximum permissible concentrations of the HMs (ppm): Cd—1.5, Cr—80.0, Hg—0.5, Mo—5.0, Pb—80.0, Zn—300.0. Number in superscript indicates times in which heavy metal concentration exceeds the limits.

**Table 2:** Polymorphic transcript-derived fragments (TDFs) detected in Co-treated and untreated *Vicia faba* plants after one month of growth in control soil and polluted soil mix from the territory of the former drill factory (DF).

Group	TDF	TDF Length, nt	Intensity of Polymorphic TDF Bands								Homologous Gene	Sequence Homology, %	E value	Accession Number
			Control Soil				DF Soil Mix							
			C	NG	LG	Y	C	NG	LG	Y				
I	N4	350	1	1 ↓	1	1 ↓	0	0	0	0	*Medicago truncatula* carboxy-terminal region remorin	96.00	2 × 10^−9^	XM_013589416.1
	N13	300	1	1 ↓↓	1	1 ↓	1	0	0	0	PREDICTED: *Medicago truncatula* cell division control protein 2 homolog	87.15	2 × 10^−52^	XM_013601820.2
	N18	200	1	1	1	1	0	0	0	0	*Lupinus angustifolius* cultivar Tanjil chromosome LG-04	77.59	0.44	CP023116.1
	N29	220	1 ↓	1 ↓	1 ↓	1	0	0	0	0	*Chionochloa rigida* subsp. *amara PsbM (psbM)* gene	90.91	0.053	GQ305171.1
	N37/N41	300	1	1 ↓	1 ↓	1	0	0	0	0	*Pisum sativum* PSI light-harvesting antenna chlorophyll a/b-binding protein (lhcA-P4)	91.44	6 × 10^−65^	AF002248.3
	N49	150	1	1	1	1	0	0	0	0	PREDICTED: *Medicago truncatula* cyclic dof factor 2	91.67	2 × 10^−9^	XM_003618459.3
	N33	240	1	1	0	0	0	0	0	0	*Pisum sativum* DEAD box RNA helicase	90.34	1 × 10^−55^	AY167671.1
II	N17	370	0	0	0	0	1	1	1 ↓	1 ↓	*Vicia villosa* chloroplast NADH dehydrogenase subunit 4-like	96.92	4 × 10^−133^	KT457043.1
	N47	320	1 ↓	0	0	0	1	1	1	1 ↓	*Medicago truncatula* trehalose-6-phosphate synthase domain protein	84.42	4 × 10^−32^	XM_003630929.3
	N54	180	0	0	0	0	1	1	1	1	PREDICTED: *Cicer arietinum* uncharacterized	86.96	3 × 10^−21^	XM_004504383.3
III	N35	440	1	1	1	1	1 ↓	1 ↓	1 ↓	1 ↓	PREDICTED: *Medicago truncatula* kunitz trypsin inhibitor 2	84.09	1 × 10^−83^	XM_003620121.3
	N36	400	1 ↓	1 ↓	1 ↓	1 ↓	1	1	1	1	PREDICTED: *Medicago truncatula* pentatricopeptide repeat-containing protein At1g71460, chloroplastic	82.20	4 × 10^−78^	XM_003604187.3
IV	N32	250	1	0	0	0	1 ↓	0	0	0	PREDICTED: *Medicago truncatula* elongation factor Tu, chloroplastic	77.91	0.001	XM_003601112.3
	N40	350	0	0	0	1	0	0	0	1	PREDICTED: *Medicago truncatula* IQ domain-containing protein IQM1	84.62	7 × 10^−8^	XM_013603017.2

Only soil-specific and phenotypic group-specific polymorphic TDFs were selected. A full list of polymorphic TDFs and their homology to known genes is presented in Table S4. Plant phenotypic groups: C—Co-untreated (control) plants; NG—normal green, LG—light green, Y—yellow phenotypes of Co-treated plants. 0, 1 ↓↓, 1 ↓ and 1 represent the intensity of an individual TDF band in ascending order.

## Data Availability

Data used to support the findings in this study are contained within this article and the Supplementary Materials.

## Supplementary Materials

The following supporting information can be downloaded at: https://www.mdpi.com/article/10.3390/antiox11040793/s1, Figure S1: Growth progress of Co-treated *V. faba* plants in DF soil mix after one (A), two (B) and three (C) weeks after sowing; Table S1: Morphometric characteristics (A), Pearson correlation matrix between plant morphological traits and soil chemistry-based indices Zs and RI (B) and eigenvalues, variance explained (%) and correlations between the principal components and the original variables (C) obtained from Co-untreated *V. faba* plants grown in soils of different pollution levels; Table S2: Frequency of morphoses (A), Pearson correlation matrix between different groups of morphoses and soil chemistry-based indices Zs and RI (B) and eigenvalues, variance explained (%) and correlations between the principal components and the original variables (C) obtained from Co-treated and untreated *V. faba* plants grown in soils of different pollution levels; Table S3: The content of biochemical nonenzymatic stress markers (A), frequencies of SOD isozyme profiles (B) and concentrations of photosynthetic pigments (C) observed in Co-treated (NG, LG and Y) and untreated *Vicia faba* plants grown in control and polluted (DF mix) soils. (D) Pearson correlation matrix for all tested biochemical parameters and (E) eigenvalues, variance explained (%) and correlations between the principal components and the original biochemical variables obtained from Co-treated and untreated *V. faba* plants from soils of different pollution status; Table S4: Full list of polymorphic transcript-derived fragments (TDFs) observed in Co-treated and untreated *V. faba* plants after one month of growth in control soil and polluted soil mix from the territory of the former drill factory (DF); Table S5: The size and number of loci obtained with functional CDDP primers used in the evaluation of Co- and soil-induced changes in the DNA sequence of *Vicia faba* plants after 1 month of exposure to the tested soils.
