# Supplementary material for: The Butterfly Effect: Mild Soil Pollution with Heavy Metals Elicits Major Biological Consequences in Cobalt-Sensitized Broad Bean Model Plants
Source: Antioxidants (Basel). 2022 Apr 18;11(4):793. doi: 10.3390/antiox11040793 (PMC9028058; doi:10.3390/antiox11040793)
Supplement: Supplementary file 1 [file antioxidants-11-00793-s001.zip › antioxidants-1684394-supplementary-done.pdf]

antioxidants

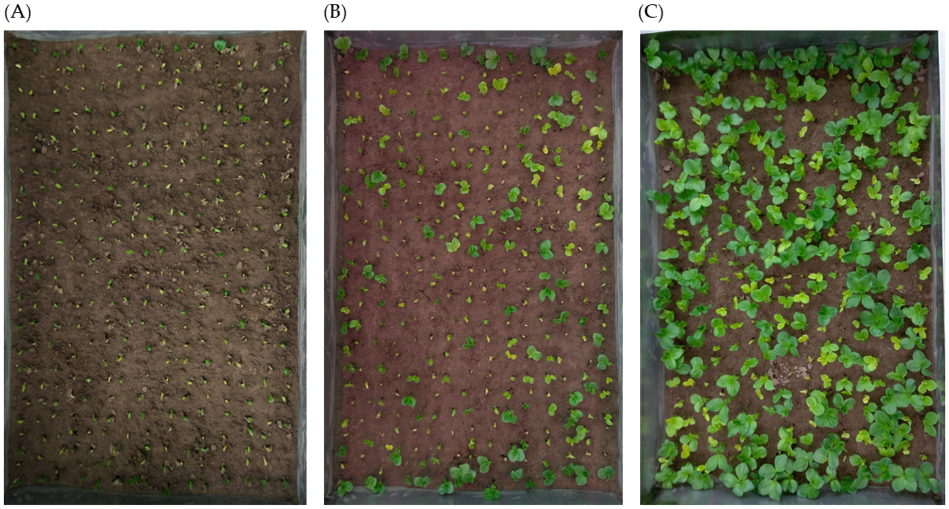

Figure S1. Growth progress of Co-treated *V. faba* plants in DF soil mix after one (A), two (B) and three (C) weeks after sowing.

Table S1. Morphometric characteristics (A), Pearson correlation matrix between plant morphological traits and soil chemistry-based indices Zs and RI (B) and eigenvalues, variance explained (%) and correlations between the principal components and the original variables (C) obtained from Co-untreated *V. faba* plants grown in soils of different pollution levels.

(A)

Soil sample	Plant height, cm	Dry weight, g	Leaf number per plant	Zs	RI
Control	27.5 ± 0.9	5.3 ± 0.3	3.8 ± 0.3	-	3.58
DF1	19.4 ± 1.5[c]	3.0 ± 0.3[c]	2.7 ± 0.2[b]	26	11.49
DF2	20.3 ± 0.9[c]	3.0 ± 0.3[c]	2.8 ± 0.1[b]	30	11.13
DF3	21.8 ± 1.6[b]	3.2 ± 0.3[c]	2.8 ± 0.2[b]	37	79.58
DF4	22.1 ± 1.2[c]	3.6 ± 0.3[c]	3.0 ± 0.2[a]	17	9.36
DF5	24.3 ± 2.0	3.2 ± 0.3[c]	3.5 ± 0.2	65	13.88
DF6	23.0 ± 1.0[c]	3.9 ± 0.3[c]	3.1 ± 0.1[a]	104	128.74
DF7	21.7 ± 1.7[b]	3.5 ± 0.3[c]	2.9 ± 0.2[a]	83	83.16
DF8	20.1 ± 2.4[b]	3.3 ± 0.4[c]	3.1 ± 0.2	67	13.94
Average of DFs	21.6 ± 0.5[c]	3.3 ± 0.2[c]	3.0 ± 0.2[a]	53.6 ± 2.1	43.9 ± 2.6

(B)

	Plant height, cm	Dry weight, g	Leaf number per plant	Zs	RI
Plant height, cm	1				
Dry weight, g	**0.8527**	1			
Leaf number per plant	**0.9302**	**0.7953**	1		
Zs	-0.1842	-0.2817	-0.1147	1	
RI	-0.0205	-0.0269	-0.1952	**0.7245**	1

(C)

	PC 1	PC 2	PC 3	PC 4	PC 5
Eigenvalue	2.8	1.6	0.4	0.1	0.01
Variance, %	56.6	32.5	8.2	2.6	0.2
Plant height, cm	0.5601	0.2040	0.0149	-0.5705	-0.5646
Dry weight, g	0.5414	0.1450	-0.4248	0.6993	-0.1284
Leaf number / plant	0.5535	0.1500	0.4773	-0.0477	0.6642
Zs	-0.2410	**0.6540**	0.5514	0.3279	-0.3193
RI	-0.1690	**0.6980**	-0.5363	-0.2751	0.3488

0.2-0.39 weak corr
0.4-0.69 moderate corr.
0.7-0.89 strong corr.
0.9-1.0 very strong corr.

DF1-8 – soil samples from territory of former drill factory; Zs – index of the total soil contamination, that accounts only for elements exceeding background level; RI – potential ecological risk index, consisting of the sum of potential ecological risk indices for all detected potentially harmful elements; [a,b,c]represent significant differences ([a]$P < 0.05$; [b]$P < 0.01$; [c]$P < 0.001$) between the control and soil collected from different DF sites. Statistically significant ($P < 0.05$) correlation coefficients are presented in bold.

Table S2. Frequency of morphoses (A), Pearson correlation matrix between different groups of morphoses and soil chemistry-based indices Zs and RI (B) and eigenvalues, variance explained (%) and correlations between the principal components and the original variables (C) obtained from Co-treated and untreated *V. faba* plants grown in soils of different pollution levels

(A)

Soil sample	n	Germination, %	Frequency of morphoses, %		
			NG	LG	Y
C	30	100	53 ± 9	40 ± 9	7 ± 5
DF1	26	90[a]	22 ± 8[c]	44 ± 10	33 ± 9[c]
DF4	28	93	29 ± 9[c]	64 ± 9[c]	7 ± 5
DF6	45	90[b]	4 ± 3[c]	56 ± 7[c]	40 ± 7[c]
DF7	25	83[c]	20 ± 8[c]	44 ± 10	36 ± 10[c]
DF8	19	95	11 ± 7[c]	68 ± 11[c]	21 ± 10[c]

(B)

	RI	Zs	Germination, %	NG, %	LG, %	Y, %
RI	1					
Zs	0.8706	1				
Germination, %	-0.6200	-0.6397	1			
NG, %	-0.6100	**-0.8520**	0.5777	1		
LG, %	-0.0620	0.2416	0.1427	-0.5334	1	
Y, %	**0.7590**	**0.7970**	**-0.7861**	-0.7349	-0.1816	1

(C)

	PC 1	PC 2	PC 3	PC 4	PC 5
Eigenvalue	3.9	1.4	0.4	0.2	0.1
Variance, %	65.3	23.0	7.1	3.7	0.9
RI	0.4400	-0.1428	0.6297	-0.3678	-0.5043
Zs	0.4798	0.1221	0.3138	-0.0452	0.8089
Germination, %	-0.4068	0.2572	0.6611	0.5752	-0.0218

0.2-0.39 weak corr.
0.4-0.69 moderate corr.
0.7-0.89 strong corr.

NG, %	-0.4400	-0.3757	0.1929	-0.3565	0.2232
LG, %	0.0621	**0.8379**	-0.0961	-0.2256	-0.1386
Y, %	0.4617	-0.2350	-0.1469	0.5950	-0.1480

n – number of tested plants; Phenotypes of morphoses: C – Co-untreated (control) plants, NG – normal green, LG – light green, Y – yellow Co-treated plants; ^{a,b,c}represent significant differences (^{a}P<0.05; ^{b}P<0.01; ^{c}P<0.001) between the control and soil collected from different DF sites. Statistically significant (P < 0.05) correlation coefficients are presented in bold.

Table S3. The content of biochemical nonenzymatic stress markers (A), frequencies of SOD isozyme profiles (B) and concentrations of photosynthetic pigments (C) observed in Co-treated (NG, LG and Y) and untreated *Vicia faba* plants grown in control and polluted (DF mix) soils. (D) Pearson correlation matrix for all tested biochemical parameters and (E) eigenvalues, variance explained (%) and correlations between the principal components and the original biochemical variables obtained from Co-treated and untreated *V. faba* plants from soils of different pollution status.

(A)

Soil-phenotype	Group I		Group II			
	H_2O_2, µM/g FW	MDA, nmol/g FW	Free proline, µg/g FW	Ascorbic acid, mg/g FW	Polyphenols, µg/g FW	Flavonoids, µg/g FW
**C-C**	5.73 ± 0.63	18.40 ± 3.56	44.40 ± 3.74	0.97 ± 0.11	63.9 ± 9.7	328.3 ± 14.3
**C-NG**	9.80 ± 0.80	13.41 ± 0.92^{1}	58.40 ± 8.80	1.55 ± 0.14	182.6 ± 25.8^{1}	489.5 ± 24.4^{1}
**C-LG**	6.50 ± 0.80	14.41 ± 2.31	76.10 ± 9.00	1.60 ± 0.19	171.2 ± 23.8^{1}	374.6 ± 26.9
**C-Y**	8.10 ± 3.20	14.87 ± 2.91	47.90 ± 2.80	1.34 ± 0.11	135.5 ± 9.8^{1}	242.6 ± 5.2^{1}
**DF-C**	5.78 ± 0.81	17.96 ± 3.88	26.85 ± 1.23^{a}	1.10 ± 0.13	68.5 ± 16.2	361.7 ± 3.9
**DF-NG**	11.80 ± 1.20	13.03 ± 1.03^{2}	31.10 ± 4.50^{a}	1.35 ± 0.13	156.5 ± 42.7	375.4 ± 14.3^{a}
**DF-LG**	4.80 ± 2.00	11.55 ± 1.92^{1}	37.90 ± 3.10^{1a}	0.98 ± 0.07^{a}	138.3 ± 50.5	352.5 ± 73.4
**DF-Y**	2.30 ± 1.10	10.66 ± 1.03^{2}	42.10 ± 5.60^{1}	0.94 ± 0.05^{b}	111.5 ± 29.9	295.3 ± 2.4^{1a}

(B)

Soil-phenotype	Types of SOD isozyme profiles			
	I	II	III	Other
**C-C**	0.33 ± 0.01	0.19 ± 0.01	0.48 ± 0.02	0
**C-NG**	0.44 ± 0.02	0.11 ± 0.01^{1}	0.33 ± 0.02^{1}	0.12 ± 0.01
**C-LG**	0.58 ± 0.02^{3}	0.25 ± 0.02	0.17 ± 0.01^{3}	0
**C-Y**	0.29 ± 0.01	0.29 ± 0.02^{1}	0.42 ± 0.02	0
**DF-C**	0.47 ± 0.02^{c}	0.16 ± 0.01	0.37 ± 0.01^{a}	0
**DF-NG**	0.38 ± 0.02	0.24 ± 0.01^{a}	0.38 ± 0.02	0
**DF-LG**	0.25 ± 0.02^{c}	0.42 ± 0.02^{3a}	0.33 ± 0.02^{b}	0
**DF-Y**	0.39 ± 0.01^{a}	0.22 ± 0.01^{1}	0.39 ± 0.01	0

Diversity of SOD profiles

I II III

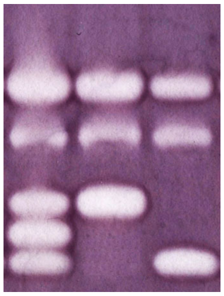

Mn SOD

Fe SOD

a b c

Cu/Zn SOD

(C)

Soil- phenotype	Group III					
	Photosynthetic pigments, mg/g FW				Pigment ratio	
	Chl a	Chl b	Chl(a+b)	Car	Chl a/b	Chl(a+b)/Car
**C-C**	1.10 ± 0.05	0.36 ± 0.01	1.46 ± 0.06	0.25 ± 0.01	3.27 ± 0.08	5.69 ± 0.07
**C-NG**	1.08 ± 0.09	0.38 ± 0.04	1.46 ± 0.13	0.27 ± 0.02	2.97 ± 0.16	5.39 ± 0.11
**C-LG**	0.42 ± 0.05^{1}	0.09 ± 0.01^{1}	0.51 ± 0.06^{1}	0.13 ± 0.01^{1}	4.98 ± 0.30^{2}	3.71 ± 0.28^{2}
**C-Y**	0.06 ± 0.01^{3}	0.02 ± 0.01^{3}	0.08 ± 0.01^{3}	0.05 ± 0.01^{2}	3.96 ± 0.66	1.65 ± 0.19^{3}

**DF-C**	1.12 ± 0.03	0.33 ± 0.02	1.45 ± 0.06	0.26 ± 0.01	3.30 ± 0.15	5.53 ± 0.04
**DF-NG**	1.13 ± 0.02	0.36 ± 0.02	1.49 ± 0.04	0.28 ± 0.01	3.24 ± 0.14	5.37 ± 0.06
**DF-LG**	0.54 ± 0.06^{1}	0.11 ± 0.01^{2}	0.65 ± 0.05^{1}	0.16 ± 0.01^{1}	5.11 ± 0.29^{2}	4.09 ± 0.26^{2}
**DF-Y**	0.11 ± 0.02^{3}	0.02 ± 0.01^{3}	0.13 ± 0.02^{3}	0.07 ± 0.01^{3}	5.45 ± 0.67^{2}	1.88 ± 0.16^{3}

Phenotypes of morphoses: C – Co-untreated (control) plants, NG – normal green, LG – light green, Y – yellow Co-treated plants. Soil types: C – control soil, DF – drill factory soil mix. MDA – malondialdehyde, Chl – chlorophyll, Car – carotenoids, SOD – superoxide dismutase, FW – fresh weight. Significance level: $^{a}P<0.05$, $^{b}P<0.01$ compared with the respective phenotypic group from control (C) soil; $^{1}P<0.05$, $^{2}P<0.01$, $^{3}P<0.001$ compared with Co-untreated plants, grown in the same soil variant.(D)

	**Chl a**	**Chl b**	**Chl a+b**	**Chl a/b**	**Chl/Car**	**Car**	**Free Pro**	**Ascorbic acid**	**H_2O_2**	**SOD profile type I**	**SOD profile type II**	**SOD profile type III**	**SOD other types**	**Polyphenols**	**Flavonoids**	**MDA**
Chl a	1															
Chl b	0.9851	1														
Chl a+b	0.9991	0.9914	1													
Chl a/b	-0.7938	-0.8643	-0.8141	1												
Chl/Car	0.9794	0.9384	0.9716	-0.6984	1											
Car	0.9968	0.9812	0.9955	-0.7728	0.9793	1										
Free Pro	-0.3223	-0.2957	-0.3197	0.2552	-0.2228	-0.3027	1									
Ascorbic acid	0.0531	0.1197	0.0682	-0.2540	0.0607	0.0912	**0.6704**	1								
H_2O_2	0.4799	0.5524	0.4968	**-0.6991**	0.4084	0.5052	0.0343	**0.6823**	1							
SOD profile type I	0.1633	0.1598	0.1651	-0.0299	0.1993	0.1796	0.5149	0.5785	0.0794	1						
SOD profile type II	-0.5215	-0.6162	-0.5487	0.6364	-0.4244	-0.5030	-0.0831	-0.2807	-0.2622	-0.5505	1					
SOD profile type III	0.1518	0.2083	0.1665	-0.4030	0.0273	0.0977	-0.6462	-0.6084	-0.0289	**-0.6939**	-0.1255	1				
SOD other types	0.3355	0.4368	0.3599	-0.4321	0.3021	0.3667	0.3288	0.4867	0.3997	0.1863	-0.5357	-0.1325	1			
Polyphenols	-0.1811	-0.1318	-0.1735	0.1286	-0.1568	-0.1130	0.5722	**0.7797**	0.5249	0.2278	0.1255	-0.6272	0.4928	1		
Flavonoids	0.6601	0.6715	0.6632	-0.4103	**0.6907**	**0.7055**	0.2408	0.4803	0.4355	0.4532	-0.4643	-0.4380	**0.7740**	0.4589	1	

0.2-0.39 weak corr
0.4-0.69 moderate corr.
0.7-0.89 strong corr.
0.9-1.0 very strong corr.

MDA	0.4900	0.4757	0.4910	-0.6157	0.4919	0.4294	-0.0924	-0.0299	0.0949	0.1770	-0.4214	0.2951	-0.1280	-0.6115	-0.0501	1

Statistically significant ($P < 0.05$) correlation coefficients are presented in bold.

(E)

	PC 1	PC 2	PC 3	PC 4	PC 5	PC 6	PC 7
Eigenvalue	7.4	4.2	1.6	1.2	0.9	0.5	0.1
Variance, %	46.4	26.1	10.0	7.7	5.9	3.0	0.9
Chl a	0.3523	-0.0929	0.0072	0.1690	0.0783	0.0182	0.1171
Chl b	0.3612	-0.0730	0.0439	0.0526	-0.0105	-0.0319	0.1374
Chl a+b	0.3555	-0.0894	0.0132	0.1402	0.0562	0.0015	0.1061
Chl a/b	-0.3215	0.0851	-0.0923	0.3836	-0.0376	-0.0188	0.2143
Chl/Car	0.3379	-0.0662	-0.0411	0.2708	0.1430	0.2026	0.1268
Car	0.3524	-0.0649	0.0367	0.2034	0.0818	-0.0060	0.0973
free Pro	-0.0566	0.3930	-0.2464	-0.1477	-0.0378	0.5724	0.5925
Ascorbic acid	0.1018	0.4217	0.0052	-0.3112	0.2445	-0.0426	-0.1578
H_2O_2	0.2230	0.1640	0.3656	-0.3447	0.3794	-0.2007	0.1135
Polyphenols	-0.0098	0.4353	0.3509	0.0042	0.0902	-0.0470	0.1154
Flavonoids	0.2707	0.2540	0.0675	0.3233	-0.2113	0.1029	-0.1513
MDA	0.1802	-0.1974	-0.4349	-0.3323	0.2515	0.4086	-0.3448
SOD profile type I	0.1032	0.2904	-0.5428	0.0647	0.0406	-0.4315	-0.0160
SOD profile type II	-0.2453	-0.0505	0.3025	0.2982	0.4670	0.3876	-0.2296
SOD profile type III	0.0318	-0.4008	0.2167	-0.3647	-0.2590	0.0173	0.3662
SOD profile type Other	0.1904	0.2503	0.2049	-0.0924	-0.5951	0.2666	-0.3920

Table S4. Full list of polymorphic transcript-derived fragments (TDFs) observed in Co-treated and untreated *V. faba* plants after one month of growth in control soil and polluted soil mix from the territory of the former drill factory (DF).

TDF number	TDF length, nt	Intensity of polymorphic TDF bands								Homologs in plants	Sequen-ce homolo-gy, %	E value	Accession number
		Control (C) soil				Drill factory (DF) soil							
		C	NG	LG	Y	C	NG	LG	Y				
N1	380	1	1	1	1	0	1	0	0	*Pisum sativum* gene for plastocyanin	98	8×10^{-123}	X16082.1
										PREDICTED: *Medicago truncatula* plastocyanin (LOC11426844)	89	4×10^{-88}	XM_003603795.3
										Glycine max plastocyanin (LOC100306554)	74.3	3×10^{-34}	M_001249724.3
N2	540	1	1	0	1	1	1	0	0	PREDICTED: *Medicago truncatula* eukaryotic aspartyl protease family protein, mRNA	87.15	8×10^{-118}	XM_003612194.3
										PREDICTED: *Cicer arietinum* aspartyl protease family protein 1-like (LOC101514975)	80.73	2×10^{-92}	XM_004512119.2
										PREDICTED: *Glycine soja* aspartyl protease family protein 1-like (LOC114419518)	79.17	1×10^{-76}	XM_028385203.1
N4	350	1	1↓	1	1↓	0	0	0	0	*Medicago truncatula* carboxy-terminal region remorin mRNA	96	2×10^{-09}	XM_013589416.1
										PREDICTED: *Cicer arietinum* remorin-like (LOC101514413), mRNA	64	1×10^{-05}	XM_004510749.2
N5	300	0	0	1	0	0	0	0	1↓	*Lupinus luteus* ABA-activated 1 protein	81.48	6×10^{-09}	KM281724.1
N10	360	1	0	1↓	1	0	0	1↓	1	*Lupinus angustifolius* cultivar Tanjil chromosome LG-12	86.67	2×10^{-04}	CP023124.1
N13	300	1	1↓↓	1	1↓	1	0	0	0	PREDICTED: *Medicago truncatula* cell division control protein 2 homolog 2 (LOC25493338)	87.15	2×10^{-52}	XM_013601820.2
										PREDICTED: *Cicer arietinum* cell division control protein 2 homolog 2 (LOC101505571), mRNA	77.24	4×10^{-16}	XM_004504677.3
										PREDICTED: *Arachis hypogaea* cell division control protein 2 homolog (LOC112792320)	77.24	4×10^{-16}	XM_020378939.1

N15	270	1	1	1↓	1	1↓	0	0	1↓	*Medicago sativa* CDC2 kinase	90	2×10^{-58}	X70707.1
										Medicago truncatula cyclin-dependent kinase mRNA	89.39	8×10^{-57}	XM_013601820.2
										PREDICTED: *Arachis hypogaea* cell division control protein 2 homolog (LOC112792320)	74.46	7×10^{-20}	XM_025835510.2
N16	380	1	1	0	1↓	1	1	0	0	*Vicia faba* plastid, complete genome	100	8×10^{-148}	KF042344.1
										Vicia villosa chloroplast NADH dehydrogenase subunit 4-like	98.98	2×10^{-143}	KT457043.1
										Medicago sativa chloroplast NADH dehydrogenase subunit 4-like	97.28	6×10^{-137}	KT457032.1
N17	370	0	0	0	0	1	1	1↓	1↓	*Vicia faba* plastid, complete genome	97.95	2×10^{-137}	KF042344.1
										Vicia villosa chloroplast NADH dehydrogenase subunit 4-like	96.92	4×10^{-133}	KT457043.1
										Medicago sativa chloroplast NADH dehydrogenase subunit 4-like	95.55	1×10^{-127}	KJ850238.1
N18	200	1	1	1	1	0	0	0	0	*Lupinus angustifolius* cultivar Tanjil chromosome LG-04	77.59	0.44	CP023116.1
										Gossypium raimondii isolate D5-4 chromosome D5_05	82.86	0.44	CP032557.1
N20	400	1	1↓↓	1↓	1↓	1	1	1↓	1↓	*Medicago truncatula* 40S ribosomal protein S20-2	81.32	2×10^{-36}	XM_003625168.3
										PREDICTED: *Cicer arietinum* 40S ribosomal protein S20-2-like (LOC101509579)	76.85	7×10^{-29}	XM_004493111.2
										PREDICTED: *Medicago truncatula* 40S ribosomal protein S20-2 (LOC25484890)	81.25	4×10^{-26}	XM_013613903.2
N21	380	1↓	0	1	0	0	0	0	0	*Medicago truncatula* zinc finger protein, GH1 protein, hydroxyproline-rich glycoprotein, and bHLH TF	83.05	2×10^{-05}	EU306659.1
N22	400	1↓	1↓	1	0	1↓	0	0	0	PREDICTED: *Medicago truncatula* DNA-directed RNA polymerase V subunit 5A (LOC11419143)	72.37	7×10^{-35}	XM_003624985.3
										PREDICTED: *Cicer arietinum* DNA-directed RNA polymerase V subunit 5A-like (LOC101509909)	68.16	3×10^{-20}	XM_004493361.3
										PREDICTED: *Cajanus cajan* DNA-directed RNA polymerase V subunit 5A (LOC109815637)	92.5	1×10^{-05}	XM_020380398.1
N26	450	1↓	0	1	0	1↓	0	1↓	0	PREDICTED: *Cicer arietinum* photosystem I chlorophyll a/b-binding protein 6, chloroplastic (LOC101494214)	82.9	1×10^{-89}	XM_004488043.3
										PREDICTED: *Medicago truncatula* photosystem I chlorophyll a/b-binding protein 6, chloroplastic (LOC11440680)	82.33	5×10^{-75}	XM_003595293.3
										PREDICTED: *Glycine soja* photosystem I chlorophyll a/b-binding protein 6, chloroplastic-like (LOC114425589)	82.31	4×10^{-26}	XM_028392529.1
N29	220	1↓	1↓	1↓	1	0	0	0	0	*Pisum sativum* ultraviolet-B-repressible protein	81.03	2×10^{-19}	AY065654.1
										Chionochloa rigida subsp. *amara PsbM (psbM)* gene	90.91	0.053	GQ305171.1
N32	250	1	0	0	0	1↓	0	0	0	PREDICTED: *Medicago truncatula* elongation factor Tu, chloroplastic (LOC11412641), mRNA	77.91	0.001	XM_003601112.3
										PREDICTED: *Cicer arietinum* elongation factor Tu, chloroplastic (LOC101508466)	77.91	0.001	XM_004501812.3
N33	240	1	1	0	0	0	0	0	0	*Pisum sativum* DEAD box RNA helicase	90.34	1×10^{-55}	AY167671.1
										PREDICTED: *Medicago truncatula* eukaryotic initiation factor 4A-15 (LOC25494213)	70.13	3×10^{-12}	XM_013603006.2
										PREDICTED: *Papaver somniferum* kelch repeat-containing protein At3g27220-like (LOC113302770)	100	2.0	XM_026551713.1
N35	440	1	1	1	1	1↓	1↓	1↓	1↓	PREDICTED: *Medicago truncatula* kunitz trypsin inhibitor 2 (LOC11420195)	84.09	1×10^{-83}	XM_003620121.3
										PREDICTED: *Cicer arietinum* kunitz trypsin inhibitor 2-like (LOC101489822), mRNA	90.42	8×10^{-55}	XM_004515002.3
N36	400	1↓	1↓	1↓	1↓	1	1	1	1	PREDICTED: *Medicago truncatula* pentatricopeptide repeat-containing protein At1g71460, chloroplastic (LOC11420489)	82.20	4×10^{-78}	XM_003604187.3

									PREDICTED: *Cicer arietinum* pentatricopeptide repeat-containing protein At1g71460, chloroplastic (LOC101513833)	84.05	3×10^{-85}	XM_027335473.1
									PREDICTED: *Vigna angularis* pentatricopeptide repeat-containing protein At1g71460, chloroplastic (LOC108328265)	79.43	5×10^{-57}	XM_017562099.1
N37	500	1	1	1	1	0	0	1↓	0	*Pisum sativum* PSI light-harvesting antenna chlorophyll a/b-binding protein (lhcA-P4) mRNA, complete cds	93.16	2×10^{-139}	AF002248.3
									PREDICTED: *Medicago truncatula* chlorophyll a-b binding protein P4, chloroplastic (LOC25490405)	86.23	2×10^{-101}	XM_013603828.2
									PREDICTED: *Cicer arietinum* chlorophyll a-b binding protein P4, chloroplastic (LOC101505314)	82.17	1×10^{-84}	XM_004513844.3
N39	350	1	1	1	1	1↓	1↓	1↓	1↓	*Pisum sativum* photosystem I chlorophyll a/b binding protein	91.44	1×10^{-94}	EF208907.1
									PREDICTED: *Medicago truncatula* chlorophyll a-b binding protein P4, chloroplastic (LOC25490405)	85.38	3×10^{-72}	XM_013603828.2
									PREDICTED: *Cicer arietinum* chlorophyll a-b binding protein P4, chloroplastic (LOC101505314), mRNA	79.15	2×10^{-55}	XM_004513844.3
N40	350	0	0	0	1	0	0	0	1	PREDICTED: *Medicago truncatula* IQ domain-containing protein IQM1 (LOC25494220)	84.62	7×10^{-08}	XM_013603017.2
									PREDICTED: *Lupinus angustifolius* IQ domain-containing protein IQM1-like (LOC109356113)	83.33	0.019	XM_019599453.1
N41	300	1	1↓	1↓	1	0	0	0	0	*Pisum sativum* PSI light-harvesting antenna chlorophyll a/b-binding protein (lhcA-P4)	91.44	6×10^{-65}	AF002248.3
									PREDICTED: *Medicago truncatula* chlorophyll a-b binding protein P4, chloroplastic (LOC25490405)	86.26	3×10^{-49}	XM_013603828.2
									PREDICTED: Cicer arietinum chlorophyll a-b binding protein P4, chloroplastic (LOC101505314)	87.5	8×10^{-51}	XM_004513844.3
N42	500	1	1	1	1	1↓	1↓	1↓	1↓	*Vicia faba* plastid, complete genome	97.14	0	KF042344.1
									Lathyrus clymenum chloroplast, complete genome	95.71	0	KJ850235.1
									Pisum sativum subsp. *sativum* isolate WL1072 chloroplast, partial genome	95.09	0	MG917089.1
N43	450	1	1	0	0	1	1	1	0	*Medicago truncatula* Ser/Thr phosphatase family, 2C domain protein mRNA	90.93	1×10^{-129}	XM_003629194.3
									PREDICTED: *Cicer arietinum* probable protein phosphatase 2C 46 (LOC101506357), mRNA	88.19	1×10^{-114}	XM_004509347.3
N47	320	1↓	0	0	0	1	1	1	1↓	PREDICTED: *Cicer arietinum* alpha,alpha-trehalose-phosphate synthase [UDP-forming] 6 (LOC101489971), mRNA	87.58	7×10^{-40}	XM_004503283.3
									Medicago truncatula trehalose-6-phosphate synthase domain protein mRNA	84.42	4×10^{-32}	XM_003630929.3
N49	150	1	1	1	1	0	0	0	0	PREDICTED: *Cicer arietinum* cyclic dof factor 2-like (LOC101490004), mRNA	79.52	2×10^{-10}	XM_004489342.2
									PREDICTED: Medicago truncatula cyclic dof factor 2 (LOC11414470), mRNA	91.67	2×10^{-09}	XM_003618459.3
N50	230	1↓	1	0	1↓	1	0	0	0	*Pisum sativum* subsp. *elatius* His5 gene for Histone H1 subtype 5, strain JI1091	87.50	0.004	FR848866.1
N51	480	0	0	1	1	1↓ ↓	1↓ ↓	1	1	PREDICTED: *Cajanus cajan* putative glutamine amidotransferase GAT1_2.1 (LOC109801873), mRNA	74.26	1×10^{-22}	XM_020363023.2
									PREDICTED: *Glycine soja* putative glutamine amidotransferase GAT1_2.1 (LOC114384863), mRNA	76.14	4×10^{-22}	XM_028344684.1
N54	180	0	0	0	0	1	1	1	1	PREDICTED: *Cicer arietinum* uncharacterized LOC101492001 (LOC101492001)	86.96	3×10^{-21}	XM_004504383.3
N56	390	0	1	0	0	1↓	0	1	0	PREDICTED: *Cicer arietinum* protein GPR107-like (LOC101492747), mRNA	74.42	1×10^{-52}	XM_004494925.3
									PREDICTED: *Vigna angularis* protein GPR107-like (LOC108327775), mRNA	88.33	9×10^{-10}	XM_017561474.1

Phenotypes of morphoses: C – Co-untreated (control) plants; NG – normal green, LG – light green, Y – yellow phenotypes of Co-treated plants. 0, 1↓ ↓, 1↓ and 1 represents the intensity of and individual TDF band in ascending order.

Table S5. The size and number of loci obtained with functional CDDP primers used in the evaluation of Co- and soil-induced changes in the DNA sequence of *Vicia faba* plants after 1 month of exposure to the tested soils.

Primer	Number of analysed loci	Size range, bp	Polymorphic loci	
			number	%
WRKY-R1	14	500-220	6	42.9
Myb2	12	250-1500	3	25.0
ERF1	19	120-2300	9	47.4
KNOX3	15	210-2000	8	53.3
ABP1-1	18	180-1950	10	55.6
Total	78	120-2300	36	46.2
Average	15.6		7.2	44.8
